# DNA methylation patterns in patients with asthenospermia and oligoasthenospermia

**DOI:** 10.1186/s12864-024-10491-z

**Published:** 2024-06-17

**Authors:** Jingdi Zhang, Xiaogang Li, Rongrong Wang, Xinxin Feng, Siyu Wang, Hai Wang, Yutao Wang, Hongjun Li, Yongzhe Li, Ye Guo

**Affiliations:** 1 Department of Clinical Laboratory, State Key Laboratory of Complex Severe and Rare Diseases, Peking Union Medical College Hospital, Chinese Academy of Medical Science and Peking Union Medical College, No.1 Shuaifuyuan, Dongcheng District, Beijing, 100730 China; 2 Department of Urology, State Key Laboratory of Complex Severe and Rare Diseases, Peking Union Medical College, Peking Union Medical College Hospital, Chinese Academy of Medical Sciences, Beijing, China; 3 Medical Science Research Center, State Key Laboratory of Complex Severe and Rare Diseases, Peking Union Medical College Hospital, Chinese Academy of Medical Science and Peking Union Medical College, Beijing, China

**Keywords:** Asthenospermia, Oligoasthenospermia, DNA methylation, Sperm

## Abstract

**Background:**

Spermatogenesis is a highly regulated and complex process in which DNA methylation plays a crucial role. This study aimed to explore the differential methylation profiles in sperm DNA between patients with asthenospermia (AS) and healthy controls (HCs), those with oligoasthenospermia (OAS) and HCs, and patients with AS and those with OAS.

**Results:**

Semen samples and clinical data were collected from five patients with AS, five patients with OAS, and six age-matched HCs. Reduced representation bisulfite sequencing (RRBS) was performed to identify differentially methylated regions (DMRs) in sperm cells among the different types of patients and HCs. A total of 6520, 28,019, and 16,432 DMRs were detected between AS and HC, OAS and HC, and AS and OAS groups, respectively. These DMRs were predominantly located within gene bodies and mapped to 2868, 9296, and 9090 genes in the respective groups. Of note, 12, 9, and 8 DMRs in each group were closely associated with spermatogenesis and male infertility. Furthermore, BDNF, SMARCB1, PIK3CA, and DDX27; RBMX and SPATA17; ASZ1, CDH1, and CHDH were identified as strong differentially methylated candidate genes in each group, respectively. Meanwhile, the GO analysis of DMR-associated genes in the AS vs. HC groups revealed that protein binding, cytoplasm, and transcription (DNA-templated) were the most enriched terms in the biological process (BP), cellular component (CC), and molecular function (MF), respectively. Likewise, in both the OAS vs. HC and AS vs. OAS groups, GO analysis revealed protein binding, nucleus, and transcription (DNA-templated) as the most enriched terms in BP, CC, and MF, respectively. Finally, the KEGG analysis of DMR-annotated genes and these genes at promoters suggested that metabolic pathways were the most significantly associated across all three groups.

**Conclusions:**

The current study results revealed distinctive sperm DNA methylation patterns in the AS vs. HC and OAS vs. HC groups, particularly between patients with AS and those with OAS. The identification of key genes associated with spermatogenesis and male infertility in addition to the differentially enriched metabolic pathways may contribute to uncovering the potential pathogenesis in different types of abnormal sperm parameters.

**Supplementary Information:**

The online version contains supplementary material available at 10.1186/s12864-024-10491-z.

## Introduction

Infertility affects approximately 10–20% of couples globally, with male factors contributing to 30–50% of these cases [1]. Asthenospermia (AS) is characterized by a decreased percentage of motile spermatozoa (sperm motility defined as below 32% progressive motility [PR] or below 40% total motility), whereas oligoasthenospermia (OAS) is characterized by a low concentration of spermatozoa and low percentage of motile spermatozoa (sperm concentration defined as below 15 million sperm per milliliter [or below 39 million sperm in the ejaculate] and sperm motility defined as below 32% PR or below 40% total motility), both of which fall below the 5th World Health Organization (WHO) manual for examination of human semen [1, 2]. Although both conditions represent the principal manifestations of impaired spermatogenesis, their exact etiology remains unclear.

Spermatogenesis is a highly regulated and complex process that is influenced by various genetic and environmental factors [3, 4]. Increasing evidence suggests that increased prevalence of genetic abnormalities was associated with decreased sperm concentration and motility [5–7]. In the past decades, several novel gene mutations have been discovered in AS and OAS, such as DNAH9, KCNU1, MNS1, GSTM1 and T1, etc [8–12]. Epigenetics, another layer of gene regulation, could strongly affect gene expression and function in both sperm function and fertilizing ability [5, 13]. Furthermore, epigenetic regulation was also changed by environmental factors [5]; the dynamic epigenome would undergo substantial changes over years, affecting disease susceptibility and determining the resulting male infertility phenotype [5, 14]. Therefore, it is important to identify epigenome-based biomarkers to assess spermatogenesis capacity in unexplained infertility, complementary to the traditional genetic biomarkers. However, differences at the genetic and epigenetic levels between the different types of impaired spermatogenesis, particularly AS and OAS, remain poorly understood.

Several data have shown that epigenetic alterations, particularly DNA methylation, play a significant role in spermatogenesis, might resulting in decreased sperm number and motility [4, 5, 15]; for instance, increased methylation of the DAZL promoter CpG island has been linked to defective human sperm in oligoasthenoteratozoospermic (OAT) men [16]. More recent study has also reported that AS patients exhibited significant hypermethylation at 2 CpG sites of IGF-2, and significant decreases in methylation at one CpG site of KCNQ1, along with three CpG sites of MEST compared to healthy controls (HCs) [17]. In OAS patients, DYDC2 and NXF3 were hypermethylated [18]. However, to date, no study has assessed epigenetic differences between AS and OAS and only a few studies have specifically investigated the differences between patients with OAS and HCs.

Previous studies have primarily focused on the aberrant methylation signature of imprinted and nonimprinted genes using the 450 K BeadChip or the bisulfite sequencing polymerase chain reaction (PCR); these studies reported the abnormally methylated genes, such as PTPRN2, RPH3AL, and APCS, etc. in oligo-/oligoastheno-zoospermic, asthenozoospermic, teratozoospermic and asthenoteratozoospermic patients [19–21]. Despite the advancements made in these studies, the complex correlation between epigenetics and sperm abnormalities remains to be comprehensively illustrated. Reduced representation bisulfite sequencing (RRBS), a cost-effective and efficient method for genome-wide methylation profiling, can offer enhanced coverage and depth for analyzing epigenetics [22].

In the present study, RRBS was performed to comprehensively investigate the global DNA methylation profiles in three distinct groups (AS vs. HCs, OAS vs. HCs, and AS vs. OAS). The study aimed to elucidate the underlying pathogenesis of spermatogenesis in AS and OAS patients, and to provide novel insights into the distinct characteristics of different spermatogenesis abnormalities.

## Methods

### Participants

Five patients each with AS and OAS and six age-matched HCs were recruited from Peking Union Medical College Hospital (PUMCH) in April 2021. The inclusion criteria for AS participants were as follows: patients with no chromosomal abnormalities, varicocele, congenital hypogonadotropic hypogonadism, chronic illnesses, history of genitourinary infections impacting fertility, or exposure to heavy metals and those who did not smoke. Any diagnosed or suspected factors that might affect reproductive health were excluded through a thorough medical history conducted by experienced urologists. The same inclusion criteria were followed for patients with OAS. These patients we enrolled had no history of hormone substance abuse or medication affecting hormone balance, and no recent acute infections. The inclusion criteria for the HC group involved no history of AS or OAS, absence of diseases known to cause male infertility, and recent childbirth. Moreover, all individuals in the HC group exhibited normal sperm morphology and sperm parameters (Table 1). The included patients with AS and OAS received the same basic traditional medical treatment before collection; however, they showed poor response to the treatment. The PUMCH ethics committee approved this study (No. JS-3112).

### Sample collection and sperm cell isolation

Semen samples were collected by masturbation after sexual abstinence for a period of 2–7 days, stored in sterile containers, and the semen analysis were conducted as per the 5th WHO guidelines [2]. The sperm cell isolation was carried out using density gradient centrifugation following the 5th WHO standard. Percoll (Sigma-Aldrich, Dorset, UK) and ×10 Human Tubal Fluid (HTF) were used to prepare an iso-osmotic gradient [23]. Briefly, discontinuous double-density gradients with two layers (2 mL 40% and 2 mL 80%) were prepared. Then, 1 mL of semen mixed with the density gradient fluid was centrifuged for 20 min at 300 g. After removing the supernatan, the collected sperm cells were washed with 5 mL of x1 HTF and centrifuged for 6 min at 200 ×g. Then this procedure was repeated once more. Finally, the supernatant sperm was stored at − 80 °C for DNA extraction.

### DNA extraction and library construction

We performed DNA isolation from frozen sperm cells using a kit (FineMag Universal Genomic DNA Extraction Kit by Magnetic Bead Method, Genfine, Beijing, China, M202). Spermatozoa precipitates with a cell count between 1 × 10^6^ and 10^7^ were resuspended in 300 μL of Buffer MDA and 20 μL of Proteinase K. Next, 300 μL of Buffer GHL was added, and the mixture was inverted 3–5 times for complete dissolution. Then, 200–500 μL of the treated sample was processed with magnetic beads to isolate high-purity DNA. The quality control of the isolated DNA was performed to ensure the purity and concentration. Finally, spectrophotometry and fluorescence were used to quantify the isolated DNA samples.

For DNA library construction, all DNA samples were subjected to RRBS library preparation using the Acegen Rapid RRBS Library Prep Kit (Acegen, Cat. No. AG0422) according to the manufacturer’s protocol. In brief, 100 ng of input DNA was digested with MspI. The DNA fragments underwent end repair, A-tailing, and cytosine ligation. Fragments ranging from 150 to 300 base pairs were gel-extracted and subjected to bisulfite treatment using the EZ DNA Methylation Gold Kit (Zymo Research). After bisulfite treatment, these fragments were then PCR-amplified to construct the final DNA libraries. The library quality was assessed using Qubit 2.0, Agilent 2100, and q-PCR for estimating insert size and concentration.

### RRBS and data processing

After comprehensive DNA library analysis, the fragments were sequenced using the Illumina NovaSeq 6000 system (Illumina, Inc., San Diego, CA, USA), with the resulting raw data being stored in the FASTQ format. Base calling was performed using Illumina Casava 1.8, and data quality was assessed and refined using FastQC and Trimmomatic. In brief, we first used FastQC v0.11.7 to perform basic statistics on the raw data. Then, the raw data was processed with the software Trimmomatic [24] v0.36. For methylation analysis, Bsmap [25] v 2.7.3 was used to aligne the clean data to the reference genome downloaded from genome website directly. After mapping to genome and generating SAM file, SAMTOOLS [26] v1.9 was used to convert SAM file to BAM file, sort and index for subsequent analysis. Then the measurement and reliability assessment of differentially methylated sites were performed using the Bsmap v 2.7.3 software. Finally, the Metilene [27] v0.2-8 software identified DMRs and calculated the average methylation levels. *P*-value was adjusted using the false discovery rate (FDR). DMRs meeting the criteria of FDR < 0.05 were considered significant differences.

### Function enrichment analysis and statistical analysis

IBM SPSS Statistics version 27.0 (IBM, Armonk, NY, USA) was used for statistical analyses. The protein–protein interaction (PPI) network was constructed using the Search Tool for the Retrieval of Interacting Genes (STRING) database (https://string-db.org/) [28], with a threshold score of 0.150. The STRING database systematically collects and integrates protein–protein interactions, encompassing both physical interactions and functional associations. Its primary aim is to provide approximate confidence levels for the associations between proteins [28]. Thereafter, Cytoscape software and its plug-in Cyto-Hubba were utilized to attain a higher degree of connectivity, in order to identify the hub gene based on maximal clique centrality (MCC) [29]. MCC is a precise predictive method for ranking essential genes or gene products, with better performance compared to other methods [29, 30]. Then we selected the top 10 node genes ranked by MCC in CytoHubba analysis. These nodes are considered to be the most important in the interaction network.

Gene Ontology (GO, http://www.geneontology.org/) [31] and Kyoto Encyclopedia of Genes and Genomes (KEGG) [32] analyses were performed using DAVID [33] (*P* value < 0.05 was considered as significant enrichment) to provide potential biological functions and pathways.

## Results

### Clinical characteristics of participants

The average age of participants in the AS, OAS, and HC groups was 33.75 ± 3.94, 30.75 ± 1.80, and 34.00 ± 1.08 years, respectively. All participants were of Han Chinese ethnicity. In the AS and OAS groups, even after administering treatment, the sperm count, sperm concentration, and sperm motility of patients in these groups remained lower than those of HCs. The clinical characteristics of the five patients with AS, five with OAS, and six HCs included in this study are shown in Table 1.

**Table 1:** Clinical characteristics of the participants

Parameter	Patients with AS (*n* = 5)	Patients with OAS (*n* = 5)	Healthy controls (*n* = 6)	Normal reference ranges
Age (years)	33.75 ± 3.94^ab^	30.75 ± 1.80^a^	34.00 ± 1.08	-
Sperm count (million)	196.26 ± 29.45^ab^	28.40 ± 8.65^a^	349.81 ± 63.74	≥ 39
Sperm concentration (million/mL)	56.19 ± 10.98^ab^	10.12 ± 1.95^a^	102.46 ± 15.35	≥ 15
Sperm motility (%)	12.93 ± 4.72^a^	12.12 ± 4.33^a^	72.40 ± 4.42	≥ 42 (PR + NP)
Progressive Motility (PR) (%) (grades a + b)	13.72 ± 17.76^a^	8.87 ± 7.50^a^	64.33 ± 8.41	30
Non-Progressive Motility (NP) (%) (grade c)	3.24 ± 3.46	5.15 ± 3.38	5.64 ± 2.30	1
Semen volume (mL)	3.46 ± 0.74	2.94 ± 0.70	3.62 ± 1.29	1.4
Sperm normal morphology (%)	5.22 ± 0.90^a^	5.05 ± 1.09^a^	10.03 ± 1.68	4

Compared with HCs, ^a^*P* < 0.05; Compared with patients with OAS, ^b^*P* < 0.05

### Global differential DNA methylation profiling

**Fig. 1:**
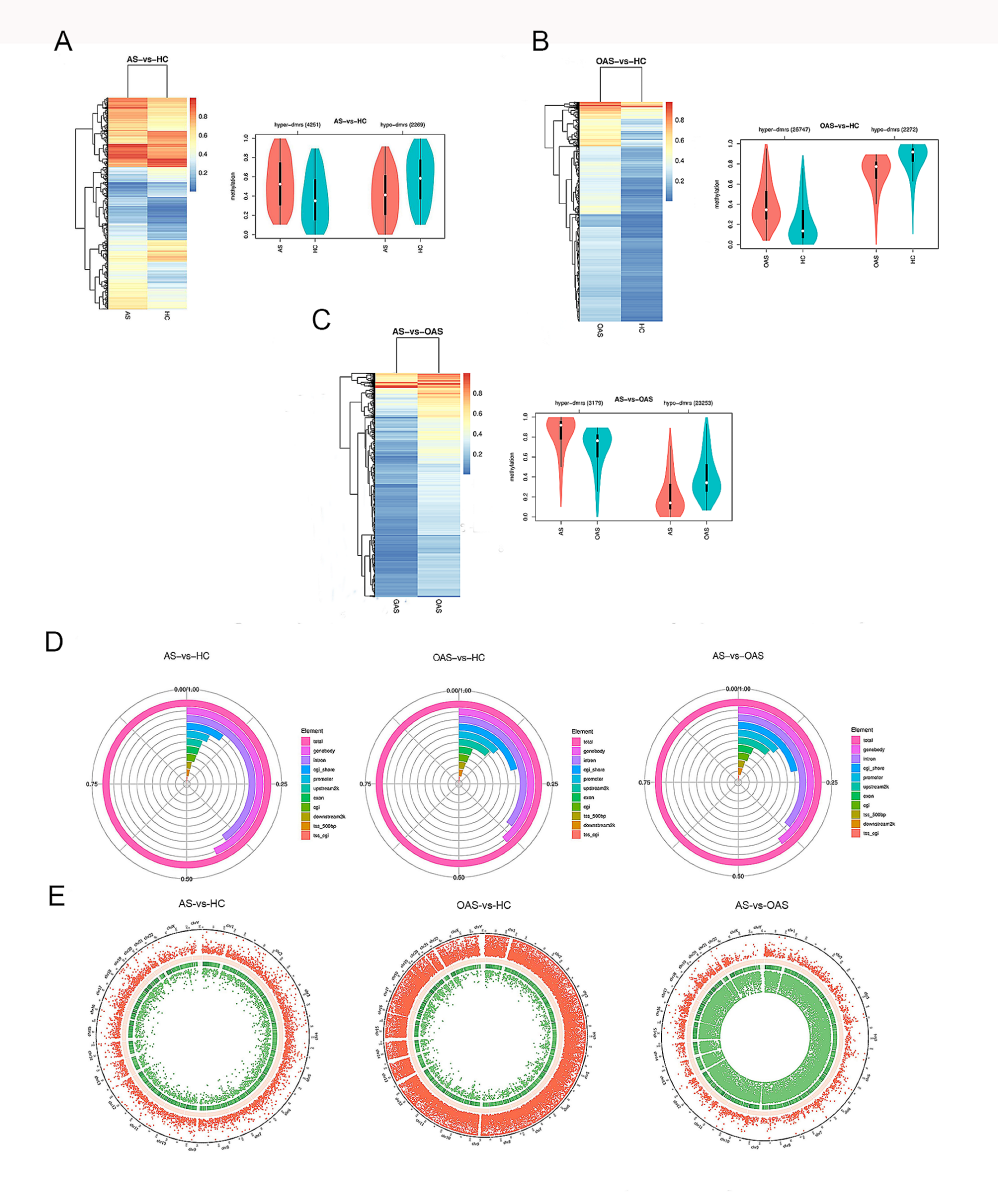
Global differential DNA methylation profiling. **A–C** DMR methylation level distribution and clustering heat map of AS vs. HC, OAS vs. HC and AS vs. OAS group. **D** DMR anchoring region distribution. **E** Circos plots of the significance of DMRs on specific chromosomes. From the outside to the inside, the distribution of hyper-DMR is shown as a scatter plot with the direction toward the outside, i.e., higher significance = more dots toward the outside; the GC content of sequences is shown as a heatmap, darker color = higher GC content; the gene density of sequences is shown as a heatmap, darker color = higher GC content; and the distribution of hypo-DMR is shown as a scatter plot with the direction toward the inside, i.e., higher significance = more dots toward the outside

To elucidate the global DNA methylation differences across various types of abnormal sperm parameters, RRBS was performed for DNA methylation profiling between three distinct pairs: AS and HCs, OAS and HCs, and AS and OAS. A total of 6520, 28,019, and 16,432 DMRs were identified between each group, respectively. Of these DMRs, the number of hypermethylated and hypomethylated DMRs was 4251 and 2269, 25,747 and 2272, and 3179 and 23,253, respectively. According to Fig. 1A, B and C, the average DNA methylation between each group was significantly different. Furthermore, the genomic zone distribution analysis of these DMRs indicated a consistent pattern across all groups, with gene bodies (44%, 39%, and 40%) being the most frequently affected region, followed by introns (40%, 36%, and 36%) and CGI shores (12%, 21%, and 22%) (Fig. 1D). The distribution of these DMRs in chromosomes is shown in Fig. 1E, which illustrates the distribution of hyper-DMRs and hypo-DMRs.

For AS and HC, OAS and HC, and AS and OAS groups, the 6520, 28,019 and 16,432 identified DMRs were mapped to 2868, 9296, and 9090 genes, respectively. The top 100 DMR-associated genes for each group are shown in additional file 1: Tables S1, S2, and S3. Among these, 56 genes were hypermethylated and 9 were hypomethylated in the AS group compared with the HC group. In the OAS and HC groups, all 58 annotated genes were hypermethylated, whereas in the AS and OAS groups, 44 genes were hypomethylated. In summary, these results suggest that the top 100 genes associated with DMRs show consistent patterns of methylation levels, both high and low, which mirror the overall methylation profiles observed across the entire genome.

### Integrative analysis and PPI network construction of DMR-associated genes

**Fig. 2:**
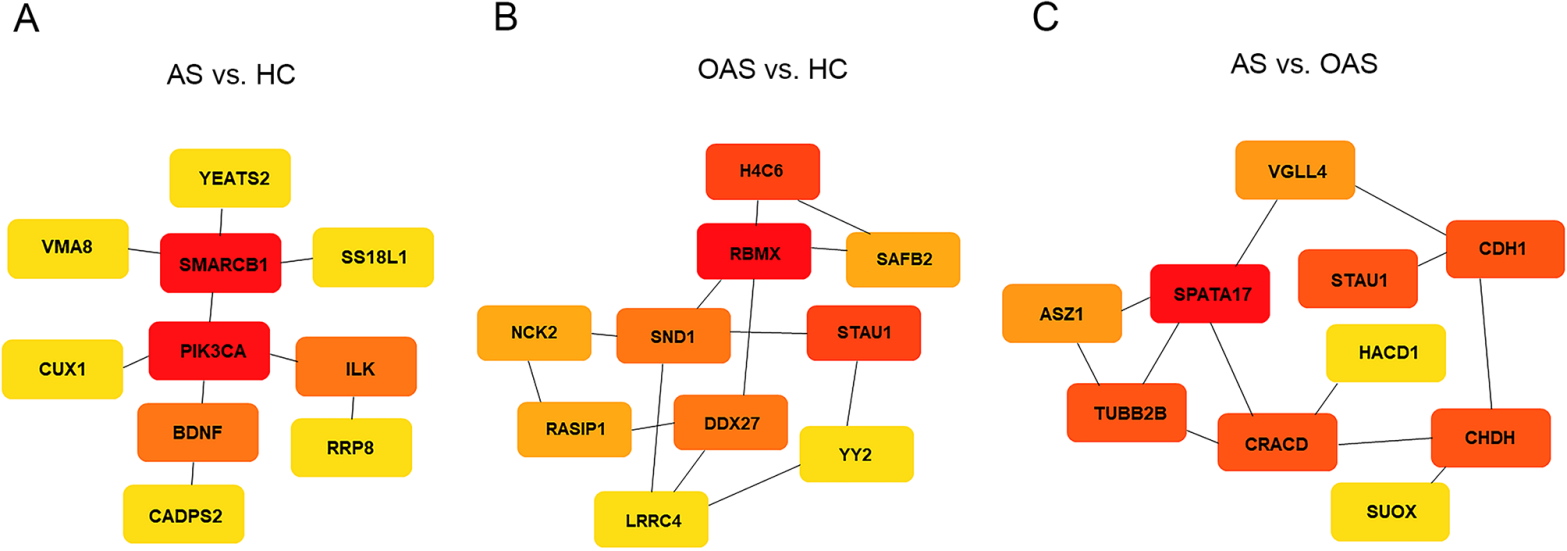
Identification of key hub genes using Cytoscape’s CytoHubba. **A**-**C** Top 10 node genes in each group based on maximum clique centrality

Genes that play pivotal roles in spermatogenesis, male infertility, asthenozoospermia, and oligoasthenospermia were comprehensively assessed using the National Center for Biotechnology Information (https://www.ncbi.nlm.nih.gov/) [34], GeneCards (https://www.genecards.org/) [35], the Online Mendelian Inheritance in Man (https://www.omim.org/) database [36], and an extensive review of current literature. The three groups included 12, 8, and 9 differential methylated genes, respectively, among the DMRs (Table 2). These genes were hypermethylated, hypermethylated, and hypomethylated in the three groups, respectively. Next, the node genes in each group were identified. In the AS vs. HC group, BDNF, SMARCB1, and PIK3A emerged as the most prominently ranked genes, whereas DDX27, RBMX, and SPATA17 and ASZ1, CDH1, and CHDH held the highest rankings in the OAS vs. HC and AS vs. OAS groups, respectively (Fig. 2A-C). Overall, the findings indicated that these differential methylated genes may be promising candidates for future research on AS and OAS.

**Table 2:** DMR-harboring genes involved in spermatogenesis or male infertility or asthenospermia or oligoasthenospermia in the AS vs. HC, OAS vs. HC, and AS vs. OAS groups

Gene	Protein	Methylation change	Function summary	Difference
**AS vs. HC**				
BDNF	Brain-derived neurotrophic factor	Hyper	Involved in male infertility	0.1601
GMCL1	Germ cell-less protein-like 1	Hyper	Spermatogenesis-associated	0.2000
SHOX	Short stature homeobox protein isoform	Hyper	Spermatogenesis-associated	0.1568
DAZL	Deleted in azoospermia like	Hypo	Spermatogenesis-associated, Involved in male infertility	-0.1548
PSMA7	Proteasome 20 S subunit alpha 7	Hyper	Spermatogenesis-associated	0.1066
MYO15A	Myosin XVA	Hyper	Involved in male infertility	0.3121
PIK3CA	Phosphatidylinositol-4,5-bisphosphate 3-kinase catalytic subunit alpha	Hyper	Spermatogenesis-associated	0.1582
POLH	DNA polymerase eta	Hyper	Involved in male infertility	0.1107
SMARCB1	SWI/SNF-related matrix-associated actin-dependent regulator of hyper chromatin, subfamily B, member 1	Hyper	Spermatogenesis-associated	0.1719
PDE4D	Phosphodiesterase 4D	hyper	Spermatogenesis-associated	0.3197
LMX1B	LIM homeobox transcription factor 1 Beta	Hyper	Spermatogenesis-associated	0.1016
GPR161	G protein-coupled receptor 161	Hyper	Spermatogenesis-associated	0.1358
**OAS vs. HC**				
DDX27	DEAD-box helicase 27	Hyper	Spermatogenesis-associated	0.4001
RBMX	RNA-binding motif protein X-linked	Hyper	Involved in asthenozoospermia and male infertility	0.3686
TSPY4	Testis-specific protein Y-linked 4	Hyper	Spermatogenesis-associated	0.4039
ING2	Inhibitor of growth family member 2	Hyper	Spermatogenesis-associated	0.3602
CRISP1	Cysteine-rich secretory protein 1	Hyper	Involved in asthenozoospermia and male infertility	0.3660
SCT	Secretin	Hyper	Spermatogenesis-associated	0.5375
INSR	Insulin receptor	Hyper	Spermatogenesis-associated	0.5822
MBTPS2	Membrane-bound transcription factor peptidase, site 2	Hyper	Spermatogenesis-associated	0.5505
PHACTR1	Phosphatase and actin regulator 1	Hyper	Spermatogenesis-associated	0.4244
**AS vs. OAS**				
DDX27	DEAD-box helicase 27	Hypo	Spermatogenesis-associated	−0.4084
CHEK2	Checkpoint kinase 2	Hypo	Spermatogenesis-associated	−0.326847
SPATA17	Spermatogenesis-associated 17	Hypo	Spermatogenesis-associated	−0.3815
ASZ1	Ankyrin repeat, SAM and basic leucine zipper domain containing 1	Hypo	Spermatogenesis-associated	−0.5298
OPRD1	Opioid receptor delta 1	Hypo	Involved in male fertility	−0.3402
CDH1	Cadherin 1	Hypo	Involved in male infertility	−0.3266
CHDH	Choline dehydrogenase	Hypo	Involved in male infertility	−0.4246
DCC	DCC netrin 1 receptor	Hypo	Spermatogenesis-associated	−0.3441

### GO and KEGG analysis of DMR-associated genes

**Fig. 3:**
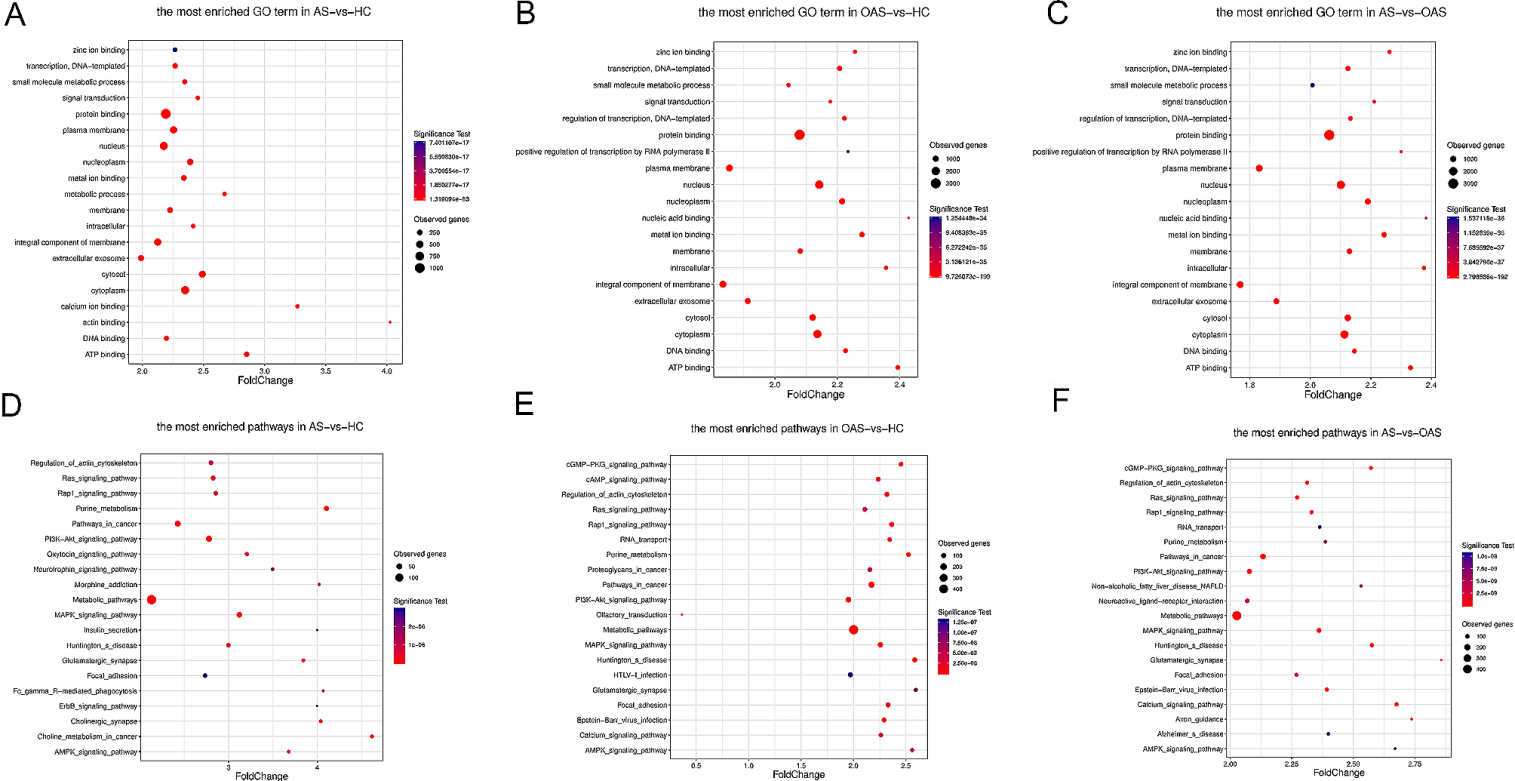
GO and KEGG annotation of DMR-associated genes. **A-C** GO analysis. **D-E** KEGG analysis

The above 2868, 9296, and 9090 genes identified from the AS vs. HC, OAS vs. HC, and AS vs. OAS groups, respectively, were subjected to GO and KEGG analyses. For the AS and HC group, GO displayed protein binding as the most enriched pathway in the biological process (BP), with cytoplasm the most enriched in the cellular component (CC) and transcription (DNA-templated) the most enriched in the molecular function (MF). Of note, similar results were obtained for both the OAS vs. HC and AS vs. OAS groups, with protein binding, nucleus, and transcription (DNA-templated) being the most enriched pathways in BP, CC, and MF, respectively (Fig. 3D-F)

### GO and KEGG analysis of DMR-associated genes in promoters

**Fig. 4:**
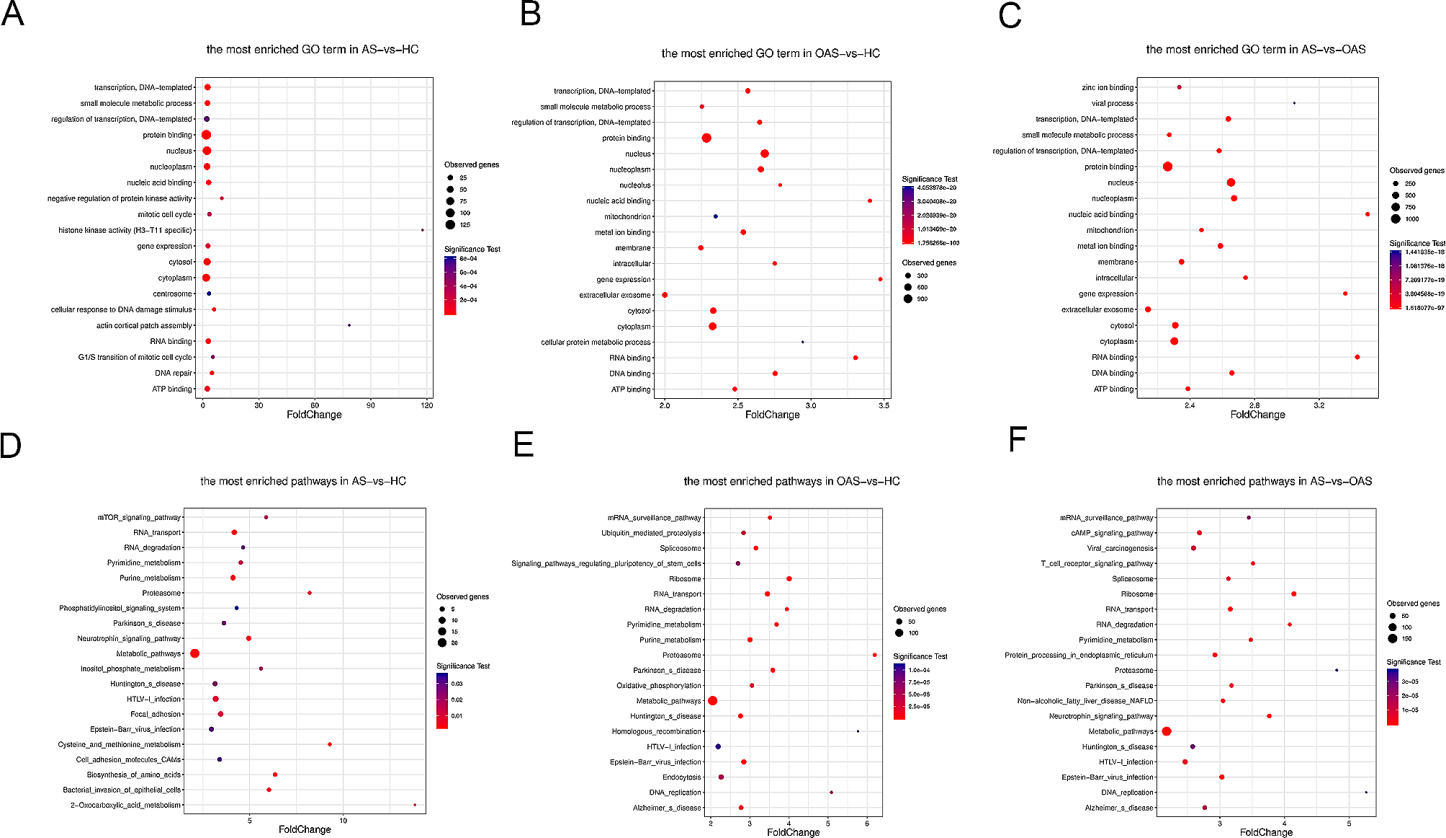
GO and KEGG annotation of DMR-associated genes in promoters. **A-C** GO analysis. **D-E** KEGG analysis

The DMR-associated genes among the AS and HC, OAS and HC, and AS and OAS groups were subjected to GO and KEGG analysis. GO results were consistent across the three groups, with protein binding emerging as the most enriched pathway in BP, nucleus in CC, and transcription (DNA-templated) in MF (Fig. 4A-C). Likewise, Fig. 4D-F shows that the metabolic pathway was the most affected pathway for all groups, in line with the results of the whole-genome analysis.

## Discussion

In our data, all HCs exhibited normal semen parameters and sperm morphology. This consistency underscores the reliability of our findings. Patients with AS and OAS demonstrated impaired sperm motility (and number), further indicating the presence of underlying mechanisms contributing to reproductive dysfunction in these patients. A comprehensive analysis also unveiled 6520, 28,019, and 16,432 DMRs differentiating patients with AS from HCs, patients with OAS from HCs, and patients with AS from those with OAS, respectively. Of note, the identified DMRs exhibited a prevalent hypermethylation pattern in both patients with AS and OAS compared with HCs. These findings align with a previous report on oligo-/oligoasthenozoospermic individuals, wherein 1436/1680 DMRs were hypermethylated [19]. The present study also revealed that these hypermethylated DMRs were primarily located within gene bodies, in line with earlier research on male infertility and sperm methylation [37–39]. Taken together, these results indicated that the DNA methylation pattern of spermatozoa may be distinct compared with that of somatic cells, challenging the conventional belief that methylation is principally enriched in the promoter region. The underlying mechanism, however, remains unknown, potentially attributed to significant differences in biological function between germ cells and somatic cells [40]. It should also be noted that sperm may undergo a complex process of epigenetic reprogramming distinct from that in somatic cells, encompassing the erasure and reconstruction of sperm epigenetic profiles [41, 42]. This intricate reprogramming may involve alterations in the dynamic equilibrium between methylation and demethylation processes throughout spermatogenesis and development, ultimately leading to distinct methylation patterns. Thus, further research focusing on DMR-associated genes located in gene bodies is needed. Of note, when patients with OAS and AS were compared, the identified DMRs contained increased hypermethylation in the former group. This indicated that a subset of samples with poorer semen parameters may harbor increased methylated alterations. A similar study reported that decreased sperm count, known as oligospermia, may be linked to modified sperm DNA methylation patterns across both imprinted and nonimprinted genes [43]. However, conflicting findings have emerged from studies investigating the correlation between DNA methylation and sperm counts. Some studies indicated no significant association, whereas others reported only select regional alterations to be associated with sperm motility [44, 45]. This discrepancy might be due to the significant heterogeneity between and within samples and owing to the small sample size analyzed in the current study.

GO analysis revealed that the identified DMR-associated genes are linked to “protein binding” in BP, in the “cytoplasm” and “nucleus” in CC, and in “transcription (DNA-templated)” in MF across the three experimental groups. An accumulation of cytoplasm has the potential to disrupt the balance of intracellular molecules, thereby triggering an elevation in the level of reactive oxygen species (ROS) [46]. Of interest, excessive levels of ROS have been reported to lead to genome-wide hypomethylation [47, 48]. They are positively correlated with IGF2 and MLH1 hypermethylation and negatively correlated with H19 hypomethylation in idiopathic male infertility, all of which are associated with semen quality and spermatogenesis interruption [48]. Furthermore, oxidative stress and DNA methylation share a common denominator: the one-carbon cycle, suggesting a significant correlation between oxidative stress, methylation processes, and epigenesis [49]. The identification of ROS-induced epigenetic damage warrants further investigation. In addition, changes in cytoplasm in the human sperm nucleus are associated with male infertility and sperm abnormalities [50]. Furthermore, the nucleus and mitochondria interact intricately, with mitochondria playing diverse roles in epigenetic regulation, gametogenesis, and reproduction [51]. In summary, these findings may explain the results observed in the GO analysis of patients with AS and OAS. Moreover, the epigenetic alterations in genes involved in both cytoplasmic and nuclear components may signify changes in the intracellular milieu in these patients. Nevertheless, this hypothesis remains speculative, and the relationship among cytoplasmic and nuclear genes and ROS levels, mitochondrial function, and sperm quality needs further study. In addition, genes participating in DNA transcription that play important roles in regulating gene expression and function may be implicated in the critical regulation of spermatogenesis. This coincides with the finding of “transcription (DNA-templated)” in the MF GO terms. Meanwhile, KEGG analysis highlighted that DMR-associated genes were associated with “metabolic pathways” in all three groups. Studies have reported metabolic alterations in the seminal plasma of patients with AS and OAS [52–55]. In line, the current study data indicated that distinct metabolic alterations may be involved in the pathology of AS and OAS. However, despite the centrality of energy metabolism in the underlying etiology of AS, few studies have identified the associated metabolic pathways. Thus, the most affected metabolic pathways associated with these DMR-annotated genes in both AS and OAS need to be explored.

A total of 12, 9, and 8 differentially methylated genes were identified in AS and HC, OAS and HC, and AS and OAS groups, respectively. These genes are closely associated with spermatogenesis and male infertility. Among these, BDNF, SMARCB1, PIK3A, and DDX27; RBMX and SPATA17; ASZ1, CDH1, and CHDH were identified as strong differentially methylated candidate genes in the three pair groups, respectively. BDNF, a member of the nerve growth factor family located in the human sperm, has been reported to participate in mitigating oxidative stress, which is important for maintaining sperm motility and facilitating sperm–egg. Therefore, alterations in BDNF may be associated with an impaired capacity of sperm to defend against oxidative stress, contributing to asthenospermia and related disorders [56, 57]. Of note, a recent investigation conducted in zebrafish models revealed that paternal exposure to microcystin–leucine arginine, a substance known to adversely affect male reproduction and disrupt the developmental trajectory of the offspring, resulted in a notable increase in the DNA methylation of the BDNF promoter within sperm, indicating that hypermethylated BDNF in AS observed in the present study might explain a potential connection with male reproductive outcomes [58]. SMARCB1, known as Snf5, is a key constituent of the SWI/SNF complex that regulates diverse cellular processes in model eukaryotes. The SWI/SNF complexes have been illustrated to potentially collaborate with certain specific transcription factors, thereby assisting in the activation of male-specific germline genes [59]. PIK3CA, a subtype of the regulatory subunit of phosphoinositide 3-kinase (PI3K), phosphorylates phosphatidylinositol and participates in the PI3K-Akt signaling pathway, the activation of which enhances the proliferation and confers anti-apoptotic properties to Sertoli cells [60]. In addition, the PI3K/Akt/mTOR signaling pathway is involved in the regulation and induction of hypoxia-inducible factor 1 alpha (HIF1α) expression; the overexpression of HIF1α has been linked to detrimental effects on testicular spermatogenesis [61]. Therefore, the identified hypermethylated genes in AS likely play a complex role in spermatogenic cells.

Compared with HCs, RBMX was hypermethylated in OAS. RBMX serves as the primary source for several autosomal retrotransposed genes, one of which is the human HNRNPGT gene (encoding the hnRNP G-T protein). The haploinsufficiency of HNRNPGT resulted in abnormal sperm production in mice [62]. In addition, a recent case report revealed that deleterious variants within the RBMXL2 gene have the potential to induce male infertility by contributing to a complete meiotic arrest, potentially by disrupting the normal trajectory of gene expression [63]. However, further research is needed to understand its role in spermatogenesis.

Between the AS and OAS groups, studies have shown that spermatogenesis-associated protein 17 (SPATA17) promoted apoptosis in the GC-1 cell line and that its overexpression mediated apoptosis in transgenic male mouse germ cells [64–67]. Regarding ASZ1, research on mice revealed that nuclear respiratory factor (NRF)-1, a key transcription factor that regulates mitochondrial biogenesis in germ cell development, exhibited a precise crosstalk with epigenetic modulation. This regulatory crosstalk directly influenced the expression of various germ cell-specific genes, particularly ASZ1, thereby interfering with germ cell proliferation and inducing male sterility [68]. ASZ1 also exhibited the ability to suppress retrotransposons and retrotransposon-driven aberrant chimeric transcripts within adenocytes via DNA methylation in the piRNA pathway, which is critical for the integrity of spermatogenesis [69]. Thus, the NRF-1–ASZ1–piRNA axis may be a promising regulatory pathway in spermatogenesis. In bovine studies, sperm-specific HMRs have been identified to target various spermatogenesis-related genes, including ASZ1 [70]. Meanwhile, CDH1 is considered a definitive marker delineating undifferentiated spermatogonia. It plays an indispensable role in orchestrating gonadotropin production within the testes through its facilitation of the migration and aggregation of primordial germ cells and somatic cells and establishing a critical regulatory nexus in the intricate processes of spermatogenesis [71]. CHDH deficiency in mice was shown to reduce sperm motility [72]. Moreover, the CHDH polymorphism rs12676 in males was associated with reduced ATP concentration in sperm [73].

Taken together, these studies highlight the roles of the identified genes in spermatogenesis and male infertility. They could act as promising candidates for in-depth studies to uncover the mechanisms of AS and OAS and explore their association with alterations in DNA methylation. However, it is not clear why these genes exhibit a more pronounced hypermethylation pattern in patients with OAS compared to those with AS. Thus, further studies are needed to explore the association between the DNA methylation of DMRs and gene expression.

There are several limitations in this study. First, the sample size was small, with no an independent validation cohort, thus the sampling bias was inevitable. While our study identified certain differentially methylated genes associated with spermatogenesis, and male infertility, the generalizability of these findings might be limited due to the restricted sample size. Our study inclined towards identifying trends in change rather than drawing final conclusions. Additionally, the small sample size limits subgroup analyses to potentially reveal different spermatogenic dysfunction with different demographic or clinical characteristics. Future studies with larger sample size are warranted to validate our findings. Second, this study lacked oligospermia controls. Therefore, the specific impact of decreased sperm count on sperm methylation status could not be clarified. Third, genes associated with DMRs located at gene bodies were not investigated. Future research using deep sequencing is essential to validate the candidate genes identified in the current study. Last, we utilized frozen sperm samples for DNA extraction. We acknowledge that frozen sperm may exhibit lower DNA integrity, viability, and motility values compared to fresh sperm [74, 75]; however, it is challenging to preserve and manage fresh sperm over the long term; and there is limited information available regarding the effects of cryopreservation on epigenetic modulation in sperm, and existing studies present contradictory results [75]. In the future studies, we intend to investigate the impact of the cryopreservation process on sperm DNA methylation.

## Conclusions

The current study findings revealed distinct sperm DNA methylation patterns among patients with AS and HCs, patients with OAS and HCs, and patients with AS and those with OAS. These DMRs could be the promising biomarkers of impaired spermatogenesis. The key DMR-associated genes identified in the study were associated with spermatogenesis and male infertility, suggesting their involvement in the regulation of sperm motility and quantity via aberrant DNA methylation alterations. These DMR-associated genes were enriched in metabolic pathways, shedding light on the different metabolism-related pathogenesis in different dys-spermatism types. These findings collectively provide novel sights into the impact of DNA methylation on sperm motility, sperm number, and male infertility. The validation of these candidate genes and differentially methylated loci using deep sequencing in large populations is necessary to explore their effect on sperm motility and number.

### Electronic supplementary material

Below is the link to the electronic supplementary material.

Supplementary Material 1

Supplementary Material 2

Supplementary Material 3

## Data Availability

The methylation sequencing data of healthy controls is available from NCBI and have been assigned BioProject accession PRJNA1117580(https://www.ncbi.nlm.nih.gov/sra/?term=PRJNA1117580). The methylation sequencing data of patients with asthenospermia and oligoasthenospermia is available from NCBI and have been assigned BioProject accession PRJNA1116754(https://www.ncbi.nlm.nih.gov/sra/?term=PRJNA1116754).

## Abbreviations

AS: Asthenospermia
BP: Biological process
CC: Cellular component
DMRs: Differentially methylated regions
GO: Gene Ontology
HCs: Healthy controls
KEGG: Kyoto Encyclopedia of Genes and Genomes
MF: Molecular function
NP: Non-progressive motility
OAS: Oligoasthenospermia
PPI: Protein–protein interaction
PR: Progressive motility
RRBS: Reduced representation bisulfite sequencing
